# The Statistical Report of King Edward's Fund

**Published:** 1913-12-13

**Authors:** 


### HOSPITAL ECONOMICS.

### II. The Statistical Report of King Edward's Hospital Fund.

In the first article (The Hospital, November 29) it was mentioned that for purposes of comparison in the statistical report the cost per occupied bed had been calculated under certain headings of expenditure.

Of these spending departments, the two most suitable for gauging economy, or want of economy, are " Provisions " and " Surgery and Dispensary." Of course, it is recognised that a variety of matters must affect expenditure under these headings, but taking it that the average number of occupied beds and the number of out-patient attendances remain about the same from year to year, and that the methods of distribution are as they should be, the main thing, one might say the only essential thing, having a serious influence is the price of goods.

In respect of " Management Expenses (Administration) " cost per occupied bed, prepared on the same lines as the rest of the figures, and averaged over the whole of the London hospitals, works out at a very small sum—between £1 and £2 per bed.

The other two departments, " Domestic " and " Salaries and Wages," are so often influenced by unusual expenditure that the figures at their surface value are unreliable material on which to base conclusions. Of the several considerations that lead us to that opinion two may be mentioned. Under " Domestic " a large renewal of furniture, bedding, or other articles may occur but once in two or three years. Under " Salaries and Wages," where an unusual number of employees of long standing leave the service within a short time and others are engaged at a commencing salary, an appreciable decrease in the total cost for the year is bound to result. Variations in expenditure such as these account for the fluctuations of a see-saw nature noticed in many instances in the returns.

The first issue of the statistical report showed that if the hospitals found to be above the average of cost on items controllable from year to year in the sixteen institutions chosen for illustration (and these represented about one-half of hospital expenditure for London) were to reduce their financial outgoings to the average, there would be an annual saving of £39,435.

Pursuant upon the issue of the first report there was a remarkable all-round fall in expenditure under certain headings. Between 1904 and 1906 (the last year in which accounts were prepared under the old system) many instances might be quoted. Suffice it to say that under the heading of " Provisions " alone a number of hospitals came down as much as £8 per bed.

Covering all the figures examined under the headings taken, it is claimed that the total annual value of the reductions increased gradually until £47,000 was reached for the year 1910. To give an idea of what £47,000 means in hospital expenditure it may be said that this sum would provide the total ordinary expenditure for one year of three hospitals of the size of the West London.

The reports for 1911 and 1912 tell another story. An increase in cost over the two years of £21,827 is shown. It is within the knowledge of most of us that many of the articles in daily use in hospitals—provisions, drugs, and other things—have risen considerably in cost during the last year or two; this fact may very well chiefly account for the turn in the tide. We think, however, it is possible that under the keen competition of a few years ago some expenditure of money was withheld and made to fall upon later years. The standard of comfort also for patients and workers has been rising for some time, and this fact makes its impression.

Let us now examine the returns of some of the hospitals in detail, aided by the tabulated figures of the statistical report and the published reports of the institutions themselves. The figures below give impressions, perhaps broadly correct, which will be somewhat modified by closer examination. The chief of these first impressions are:—

1. Hospital A is doing considerably less work than Hospital B at a cost of 25 per cent. more.
2. At practically equal cost Hospital C supports twenty-nine more beds than Hospital A, but treats only three-quarters as many out-patients.
3. On an annual expenditure of £1,300 less Hospital D supports sixty-four more beds than Hospital C, but treats only two-thirds of the number of out-patients.

The statistical report is full of examples such as these. And the report gives us the opportunity of examining the figures without the perplexity caused by the contrary conditions that hampered our preliminary criticism. The cost of in- and out-patients being separated, we can with the help of the tables, assisted by reference to the published reports of the hospitals, review the two departments in greater comfort. Under this light we will return to the comparisons set forth above.

1. The dearer Hospital A is an older charity than the cheaper Hospital B. Tradition, always a factor in questions of economy, taking years to overcome, may in this case account for much. Hospital A has a medical school and a laundry; allowance has been made for these features before the preparation of the averages quoted. Whether a hospital so equipped stands in a relatively favourable or unfavourable position we shall discuss more fully later; in the meantime it may be said that these conditions, however carefully figures are prepared, have a tendency to augment actual general cost.

But it takes a great deal to justify the difference between £30 and £18 per occupied bed for " Provisions." Turning to the gross cost of articles, one finds startling comparisons—£1,119 for meat, to take an item, as compared with £571: after remembering every relevant factor, and bearing in mind that the lowest in cost is not always the most economical, there is a striking difference. But it must be noticed that Hospital B does not hold its own in every particular; under " Surgery and Dispensary " its cost is lower by 16s. a bed; and small though this sum sounds, it is really representative of much discrimination in buying and care in distribution. Under " Management (Administration)," in addition, Hospital A is lowest of three of the institutions quoted in the table.

It will be noticed that the cost per out-patient attendance is 4d. higher in " A " than in " B." This sum dissected under the headings of expenditure shows " A " to be more expensive on all scores, even in the matter of " Surgery and Dispensary," where it was cheaper in the wards. We learn from the report that the number of surgical beds in these two institutions is the same.

2. Hospital A regarding itself beside Hospital C derives little consolation. " A " scores again in respect of " Surgery and Dispensary," but is higher (though not so much so as in the last comparison) under the same heading in the out-patient department.

3. Hospital D is an old building in which the wards are, we think, smaller on an average than any hospital in London. This fact would lead one to expect to find an unusually large staff of nurses employed. Yet the cost per occupied bed, " Salaries and Wages," and all items is much lower than any of the other examples. The total cost of the nursing service is nearly £300 less than a hospital with ninety-three fewer beds.

As there is such a thing as spending too little as well as spending too much, the hospitals compared can usefully ask themselves not only the question " Why is our cost so high under this item? " but also " Is our cost high enough? "

It is suggested that the lines on which inquiries might be made by the institutions chosen for comparison are such as the following:—

*Hospital A.*—Why is the work generally carried out at such great expense? Is the division of expenditure between in- and out-patients correctly calculated, especially in respect of " Surgery and Dispensary "? Are the prices paid for provisions and coal unduly high, and is there no waste in distribution? Is the lighting of the hospital properly regulated, and is electric current for medical purposes improperly charged to fuel and lighting?

*Hospital B.*—Why is the work generally done so cheaply, and is efficiency in any way sacrified to cutting down expenditure? Are the ordinary articles of food, such as meat, bread, butter, and milk, of a reasonable standard of quality? Have the drug bills been compared with prices paid elsewhere, and is care exercised in the distribution of surgical stores and the dispensing of medicines? In view of the low cost per out-patient attendance, is the division of cost under " Surgery and Dispensary " accurately made?

*Hospital C.*—Why is the cost under " Salaries and Wages " so high? Is the staff unnecessarily large? Quite a liberal amount of expenditure is allocated to out-patients, yet the cost per bed under " Surgery and Dispensary " is very high. Are right prices paid for drugs, etc., and is distribution properly regulated? Why is so much coal used?

*Hospital D.*—Having regard to the safety and proper treatment of the patients, is the nursing staff adequate? Are the drugs of the best quality, and is the precaution of an independent test ever taken?

Quite a number of interesting points arise, and in the study necessary to their elucidation much valuable knowledge will be gained. This, we take it, is the whole object of the report; it does not intend to pillory any institution; it does not suggest that one work is necessarily too expensively or another too cheaply carried out; the one desire is that constant vigilant inquiry shall be made into the common features of everyday life.

| Special Conditions. | Hospital A. General Hospital, Central London. Building not up to date. Medical School. Own Laundry. | Hospital B. General Hospital, Out-lying. Building not up to date. | Hospital C. General Hospital, three miles from Charing Cross. Modern building. | Hospital D. General Hospital, Outskirts of London. Old building, very small wards. |
|---|---|---|---|---|
| Beds occupied ... ... ... ... ... | 141 | 147 | 170 | 234 |
| Average stay of patients ... ... ... | 22.7 | 22.44 | 26.57 | 43.4 |
| Total new out-patients ... ... ... ... | 31,795 | 45,171 | 23,484 | 15,747 |
| Attendances ... ... ... ... ... | 109,211 | 161,009 | 84,107 | 73,676 |
| Cost per occupied bed under main headings: | | | | |
| Provisions ... ... ... ... ... | £30 19 2 | £18 18 6 | £24 5 2 | £21 7 0 |
| Surgery and dispensary ... ... ... | 6 8 4 | 7 12 2 | 8 15 9 | 6 5 1 |
| Domestic ... ... ... ... ... | 23 10 5 | 21 17 8 | 20 19 3 | 13 14 8 |
| Salaries and wages (maintenance) ... | 27 1 9 | 19 19 10 | 26 0 1 | 15 13 4 |
| Management (administration) ... ... | 14 1 | 18 10 | 1 4 6 | 12 10 |
| Total cost per bed on expenditure controllable from year to year ... ... ... | 88 13 9 | 69 7 0 | 81 4 9 | 57 12 11 |
| Cost per out-patient attendance ... ... | 9.06 | 5.02 | 7.33 | 6.30 |
| Specimens of expenditure: | | | | |
| Meat ... ... ... ... ... ... | *1,119 8 10 | 571 8 7 | 1,140 18 3 | 1,589 0 4 |
| Milk ... ... ... ... ... ... | *1,004 10 10 | 721 2 5 | 826 13 8 | 1,046 17 0 |
| Drugs, etc. ... ... ... ... ... | 1,015 1 3 | 1,349 0 8 | 1,275 0 9 | 883 12 0 |
| Dressings, etc. ... ... ... ... | 818 13 11 | 743 17 5 | 744 1 9 | 480 18 8 |
| Coal ... ... ... ... ... ... | *1.300 5 10 | 481 4 9 | 920 2 8 | 748 19 11 |
| Gas ... ... ... ... ... ... | *475 9 6 | 499 6 10 | 347 18 8 | 359 3 1 |
| Electric current ... ... ... ... | *549 4 10 | 187 18 1 | 328 12 0 | 279 10 11 |
| Medical salaries ... ... ... ... | 668 10 0 | 194 17 0 | 608 15 4 | 397 12 11 |
| Nursing salaries ... ... ... ... | 1,661 10 1 | 1,546 10 9 | 1,643 5 11 | 1,364 7 7 |
| Total ordinary expenditure ... ... ... | 20,064 1 8 | 16,052 6 2 | 20,089 1 1 | 18,734 18 10 |

* £140 of total provisions and £265 for fuel, power, and light are chargeable to laundry account.

